# Deep learning in ultrasound tongue imaging: a systematic review toward automated detection of speech sound disorders

**DOI:** 10.3389/frai.2025.1631134

**Published:** 2025-09-24

**Authors:** Saja Al Ani, Joanne Cleland, Ahmed Zoha

**Affiliations:** ^1^James Watt School of Engineering, University of Glasgow, Glasgow, United Kingdom; ^2^School of Psychological Sciences and Health, University of Strathclyde, Glasgow, United Kingdom

**Keywords:** articulatory imaging, child speech therapy, deep learning, motion modelling, self-supervised learning, speech sound disorders, tongue contour segmentation, ultrasound tongue imaging

## Abstract

**Background:**

Speech sound disorders (SSD) in children can significantly impact communication and development. Ultrasound tongue imaging (UTI) is a non-invasive method for visualising tongue motion during speech, offering a promising alternative for diagnosis and therapy. Deep learning (DL) techniques have shown great promise in automating the analysis of UTI data, although their clinical application for SSD remains underexplored.

**Objective:**

This review aims to synthesise how DL has been utilised in UTI to support automated SSD detection, highlighting the advancement of techniques, key challenges, and future directions.

**Methods:**

A comprehensive search of IEEE Xplore, PubMed, ScienceDirect, Scopus, Taylor & Francis, and arXiv identified studies from 2010 through 2025. Inclusion criteria focused on studies using DL to analyse UTI data with relevance to SSD classification, feature extraction, or speech assessment. Eleven studies met the criteria: three directly tackled disordered speech classification tasks, while four addressed supporting tasks like tongue contour segmentation and tongue motion modelling. Promising results were reported in each category, but limitations such as small datasets, inconsistent evaluation, and limited generalisability were common.

**Results:**

DL models demonstrate effectiveness in analysing UTI for articulatory assessment and show early potential in identifying SSD-related patterns. The included studies collectively outline a developmental pipeline, from foundational pre-processing to phoneme-level classification in typically developing speakers, and finally to preliminary attempts at classifying speech errors in children with SSD. This progression illustrates significant technological advances; however, it also emphasises gaps such as the lack of large, disorder-focused datasets and the need for integrated end-to-end systems.

**Conclusion:**

The field of DL-driven UTI assessment for speech disorders is developing. Current studies provide a strong technical foundation and proof-of-concept for automatic SSD detection using ultrasound, but clinical translation remains limited. Future research should prioritise the creation of larger annotated UTI datasets of disordered speech, developing generalisable and interpretable models, and validating fully integrated DL-UTI pipelines in real-world speech therapy settings. With these advances, DL-based UTI systems have the potential to transform SSD diagnosis and treatment by providing objective, real-time articulatory feedback in a child-friendly manner.

## 1 Introduction

Human speech enables complex communication, and challenges in articulating clear speech can negatively impact a child’s academic, social, and future employment prospects (McFaul et al., 2022). Speech sound disorders (SSD) are characterised by difficulty acquiring the spoken language’s phonemes, varying from minor issues with the articulation of one or two consonants to speech that is predominantly unintelligible. In many cases, SSDs have unidentified origins, such as cleft lip and palate, and may arise from particular challenges in other domains, including speech perception and motor production (McLeod and Baker, 2017). Untreated SSD can result in avoidance practices that damage social connections and restrict an individual’s ability to engage fully in social activities (McCormack et al., 2009). SSD affects a significant portion of the paediatric population, with over 25% of children in the UK exhibiting indications of speech-related difficulties. Approximately 3–4% of these individuals encounter enduring challenges that may remain throughout adulthood (Wren et al., 2016). A delayed diagnosis or absence of early intervention may lead to permanent educational and social disadvantages (Shahin et al., 2015).

The present assessment and treatment of SSD depend heavily on expert perceptual judgments by speech-language therapists (SLTs). However, there is a global shortage of SLTs, and increasing caseloads limit the availability of individualised therapy. This shortage has spurred interest in technology-assisted options for diagnosis and treatment (Leinweber et al., 2023). Several interactive programs have been developed for children with SSD, including Apraxia World (Wren et al., 2016), Tabby Talks (Shahin et al., 2015) Speech Training Assessment and Remediation (STAR) (Bunnell et al., 2000). These tools transform therapeutic activities into interactive games or offer automatic feedback for practising phonetic sounds. Although these applications can enhance children’s motivation and complement therapy, the majority concentrate on providing or prompting speech practice rather than conducting a thorough analysis of speech errors. Among the current systems, only a limited number employ automatic speech analysis to deliver feedback, and even those analyses exhibit restricted accuracy with disordered speech. The dependability of existing automated speech evaluation methods is inadequate for clinical use, underscoring the necessity for more robust methodologies.

**Figure 1:**
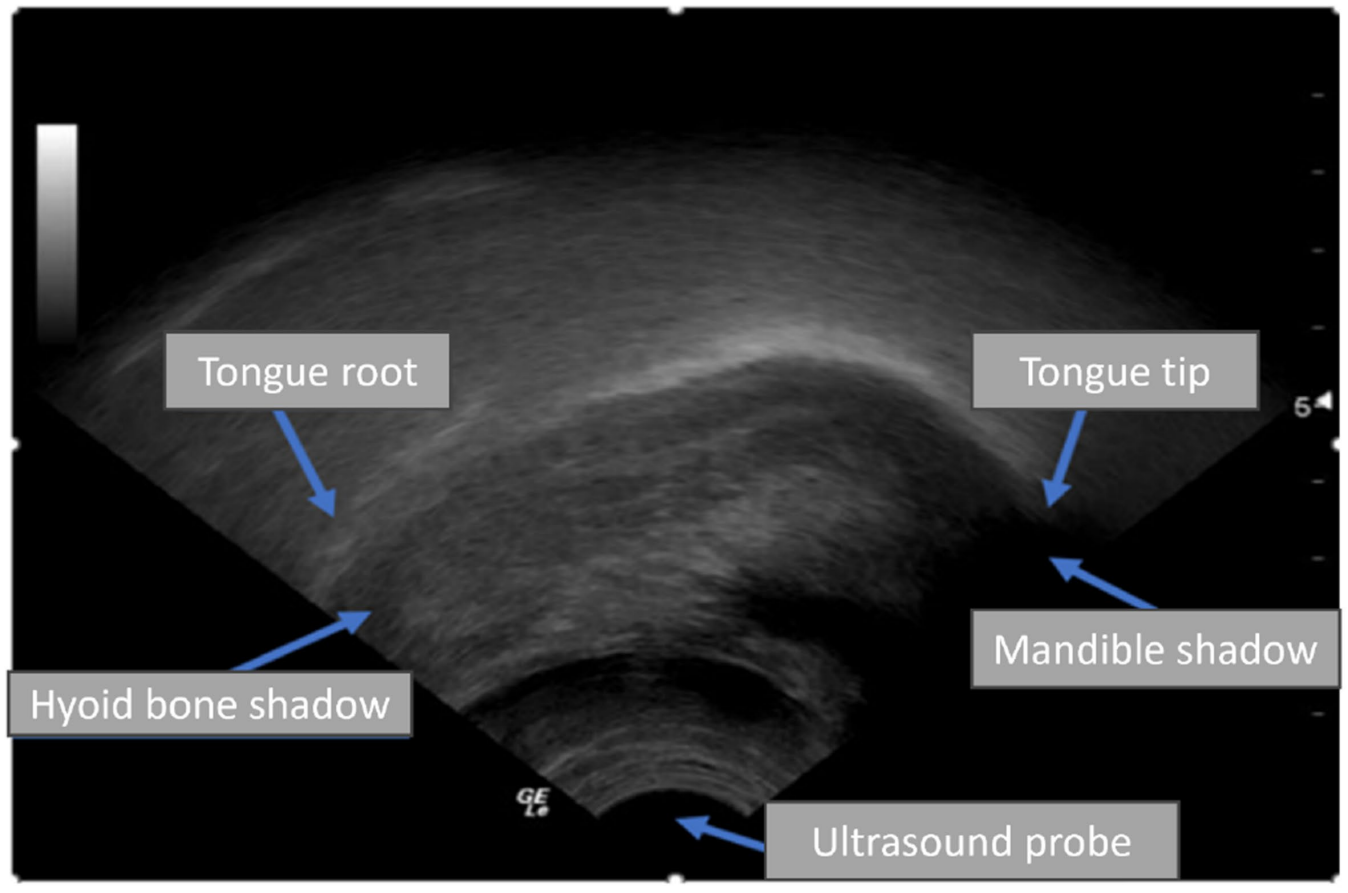
An ultrasound scan shows the tongue root and tip in the sagittal plane.

Ultrasound tongue imaging (UTI) has emerged as a viable technique to improve the diagnosis and treatment of SSD. UTI employs a probe positioned beneath the chin to capture real-time midsagittal images of the tongue during speech, facilitating the imaging of tongue shape and motion without radiation or invasiveness (Cleland, 2021; Smith et al., 2023; Hu et al., 2023). UTI is safe, suitable for children, and comparatively portable, making it appealing for paediatric speech therapy. Figure 1 illustrates a typical UTI, where the tongue surface appears as a bright arc against a darker background, with shadows produced by the hyoid bone and jaw. Clinicians and researchers have utilised UTI to deliver biofeedback in therapy and to investigate articulation, especially in those with speech disorders like childhood apraxia or cleft palate, by examining tongue patterns that are not externally observable (Preston et al., 2017). Interpreting ultrasound images traditionally requires manual tracing of tongue contours or professional analysis, both of which are time-intensive and impractical for implementation in every therapy session.

The initial computational method for analysing UTI in tongue motion tracking depended on conventional image processing (Stone, 2005). For example, EdgeTrak by Li et al. (2005) presented an active contour model for automatic frame-by-frame tracking of the tongue surface. This approach is effective when the tongue surface is identifiable, although it possesses significant limitations. EdgeTrak is deficient in advanced preprocessing capability and encounters difficulties with poor image quality or when the tongue surface is partially obscured. It is also incapable of handling extended video records without manual intervention, making it impractical for continuous speech or real-time use. Moreover, EdgeTrak’s foundational method can be computationally intensive, relying on iterative optimisation that is difficult to run in real time. These limitations indicate that although systems such as EdgeTrak demonstrated the viability of automatic tongue contour tracking, they did not entirely satisfy the requirements of interactive clinical applications (Tang et al., 2012).

Recent advancements in deep learning (DL), particularly convolutional neural networks (CNN), have facilitated automation in image segmentation (Ronneberger et al., 2015), motion tracking (Adžemović, 2025), and phoneme detection from UTI data. Nevertheless, the majority of these models are trained on data from typically developing speakers and concentrate on silent speech interfaces or language learning tasks rather than clinical SSD assessment. The variability in speaker anatomy, image quality, and dataset size continues to pose a significant obstacle to the generalisability of these systems. The publication of the UltraSuite corpus (Eshky et al., 2018), which encompasses disturbed child speech data, represents a significant advancement; yet, comprehensive assessments of DL methodologies in this clinical setting are still limited.

While a recent review by Xia et al. (2024) surveyed machine-learning techniques for UTI more broadly, there remains a need for a focused synthesis on DL methods that target clinically meaningful error detection and assessment, and on how close these approaches are to practical use in speech-language pathology.

For clinicians, DL–UTI systems can turn ultrasound videos into usable measures: flagging likely misarticulations, providing real-time visual feedback during therapy, and producing simple progress graphs across sessions. This can reduce subjectivity, focus practice on the most informative targets, and save time by rapidly screening many patients. These tools are designed to support, not replace, clinical judgement; outputs should be interpretable and integrated into routine workflows, with the clinician retaining final decision-making. This systematic review evaluates current research at the intersection of UTI and DL for SSD, tracing the field’s progression from foundational techniques, such as segmentation and motion modelling, to phoneme/gesture classification in typically developing (TD) speech, and early studies targeting direct error detection in disordered speech. We address three research questions: (1) How have DL models been applied to UTI to aid the detection or assessment of SSD? (2) What technical and clinical challenges have emerged, and how are they being addressed? (3) What advances are needed to reach clinically viable, automated UTI-based assessment and therapy support for SSD?

**Figure 2:**
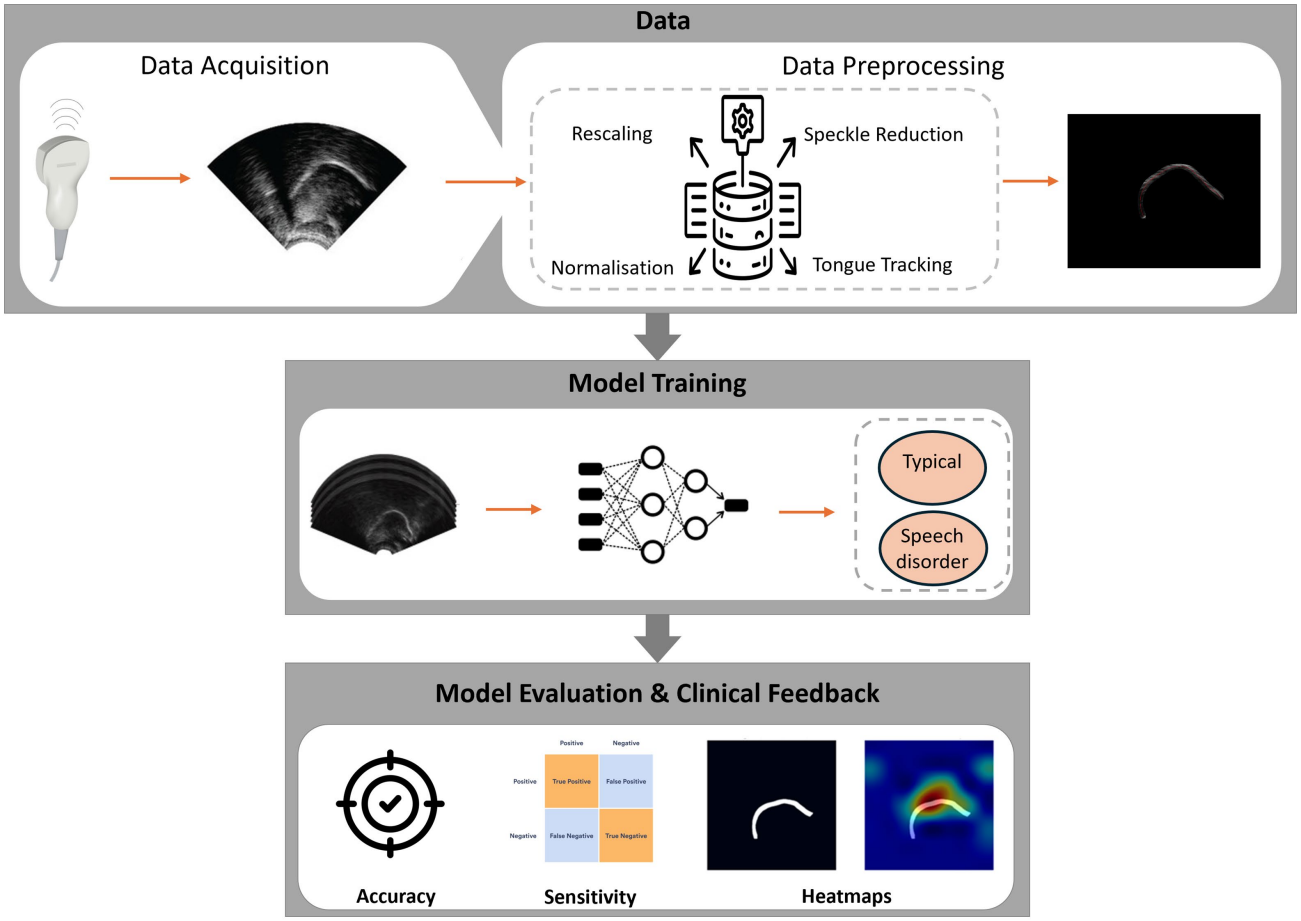
Overview of DL for UTI in SSD assessment.

To complement the scope and research questions outlined above, Figure 2 provides a high-level overview of how DL can be applied to UTI for SSD assessment, from acquisition and preprocessing through task-specific modelling to clinician-in-the-loop feedback.

The remainder of this article is structured as follows. Section 2 outlines the literature search strategy, inclusion criteria, and data extraction process (following PRISMA guidelines). Section 3 describes the included studies, organised by their contributions to a envisioned DL-based pipeline for SSD detection. Section 4 interprets and discusses the findings in context, including challenges and future research directions for advancing the field. Section 5 concludes the review by summarising the insights and translational implications, emphasising how the field can progress toward reliable, automated SSD detection using UTI and DL.

## 2 Methods

### 2.1 Aim and research questions

This comprehensive review analyses the application of DL approaches to UTI for tasks related to the detection and assessment of speech sound disorders. We place particular emphasis on the clinical relevance of these approaches and their potential for integration into speech-language pathology workflows. The review was guided by three primary research questions:

- RQ1: How have DL models been utilised in UTIs to support the detection of speech sound disorders?
- RQ2: What are the primary technical and clinical challenges that restrict the current applications of DL in ultrasound-based SSD detection?
- RQ3: What future research is necessary to progress toward clinically viable, automated UTI-based diagnosis or therapy for SSD??

### 2.2 Screening

A comprehensive literature search was conducted; our search was restricted to English-language publications across six databases: IEEE Xplore, PubMed, ScienceDirect, Scopus, Taylor & Francis Online, and arXiv. While arXiv was included to partially mitigate publication bias, we did not systematically search other grey literature sources, and we did not translate non-English reports. These choices may introduce language and database coverage bias and could underrepresent null or negative findings.

The initial search was performed in 2022 and was updated periodically through August 2025 to capture the most recent developments in this evolving field. Search queries combined keywords related to speech disorders, UTI and DL. For example, we used Boolean strings such as: “speech sound disorder” AND “ultrasound tongue imaging” AND (deep learning OR neural network), “phoneme classification” AND (ultrasound OR tongue) AND (CNN OR LSTM),” “articulatory disorder” AND “ultrasound” AND “machine learning.”

We also included synonymous terms and variations such as “speech impairment,” “convolutional,” and “articulation disorder.” the results to English-language.

After removing duplicate records, we found 112 unique publications. We performed an initial screening of titles and abstracts to exclude irrelevant papers. At this stage, 42 records were excluded because they did not relate to both ultrasound and DL. We retrieved the full text of the remaining 11 articles for detailed evaluation. Each article was assessed against the inclusion criteria described below.

### 2.3 Inclusion and exclusion criteria

Studies were included in the final review if they met all of the criteria summarised in Table 1.

**Table 1:** Inclusion and exclusion criteria.

No.	Inclusion criteria	Exclusion criteria
1	Applied a DL method to UTI data.	Focused exclusively on acoustic or other non-UTI modalities.
2	Addressed speech-related tasks such as classification, contour extraction, or motion tracking.	Used only traditional (non-DL) image processing techniques.
3	Involved human subjects (typically developing children or children with SSD).	Used ultrasound for non-speech purposes.
4	Published in English from 2010–2025, in a peer-reviewed venue.	

### 2.4 Data extraction and classification

**Figure 3:**
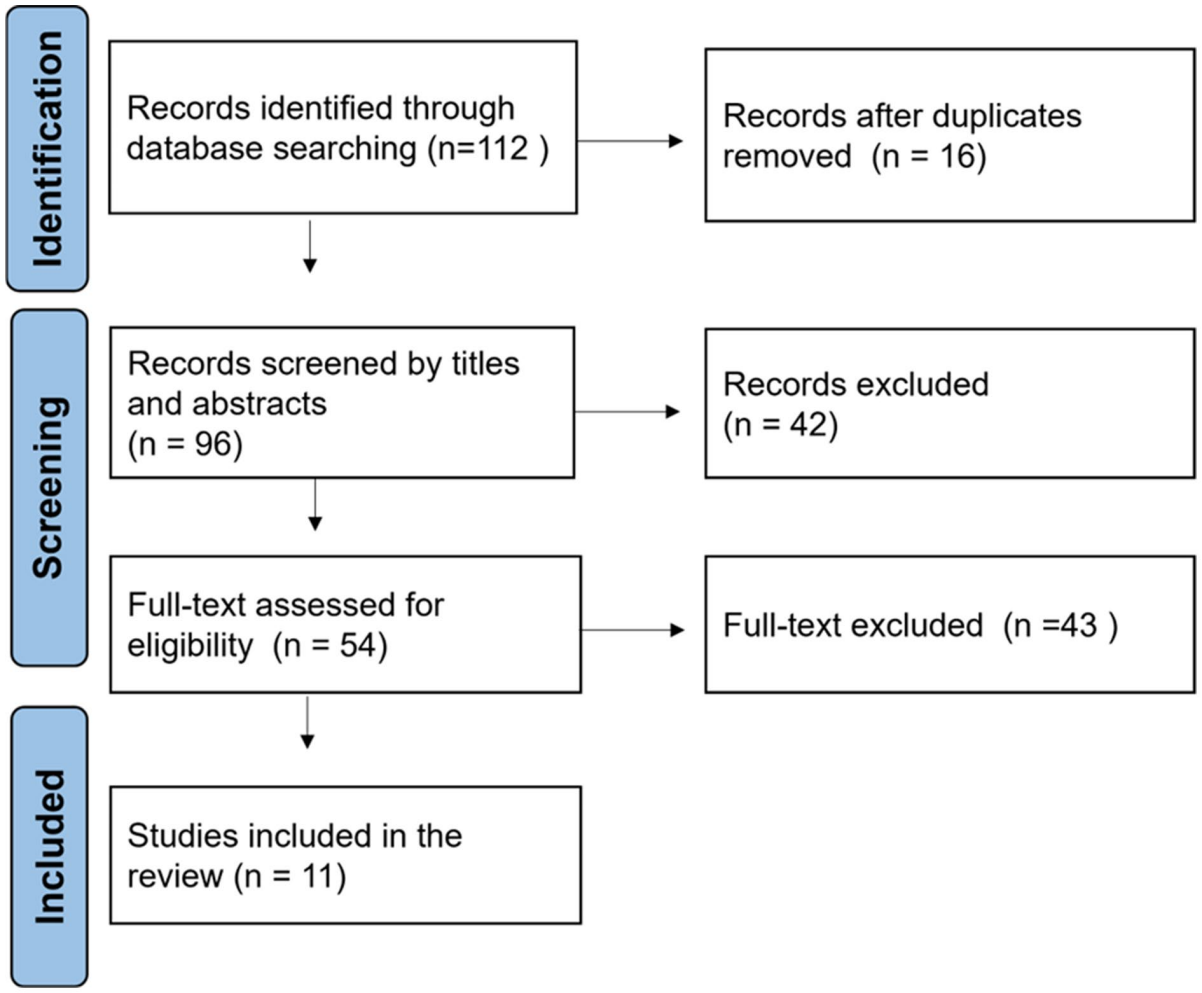
PRISMA flow diagram of the study selection process.

Following PRISMA guidelines, we documented the study selection process in a flow diagram, as shown in Figure 3. For each of the eleven included studies, we extracted key data points: the study’s title and year, the used DL model, the task, the input data type, the dataset, evaluation metrics, and any information regarding the study’s relevance to SSD diagnosing.

To synthesise the contributions of these diverse studies, we organised them into three broad categories according to their role in an envisioned end-to-end pipeline for automated SSD detection:

1. Direct SSD detection: studies that explicitly target the classification of speech sound disorder.
2. Technical foundations: studies that develop core components required for automated analysis, such as tongue segmentation or tongue motion prediction, which support the overall purpose of SSD detection.
3. Clinical context: studies that explore the use of UTI in real clinical settings or provide insight into how UTI-based feedback can be used in therapy for SSD.

This classification presents a framework for determining how each study adds to the overarching goal of establishing a fully automated and therapeutically usable system. It also helps to illustrate development by highlighting the path from basic technological advancements to direct applications and, finally, implementation considerations. Table 2 provides an overview of the included studies categorised by contribution type, summarising their methods, data, and findings regarding SSD.

**Table 2:** Summary of included studies categorised by contribution type.

Study (Year)	DL method	Dataset	Input type	Task	Metric	Technical strengths	Clinical relevance
Ribeiro et al. (2021)	CNN	SSD + Typically developing (TD) (UltraSuite)	Raw UTI + audio	Speech classification (SSD)	Accuracy, precision, recall, F1	End-to-end from raw image to error class	High – directly targets SSD classification
Ani et al. (2024)	FusionNet (CNN)	TD (UltraSuite)	Raw UTI + texture features	Phonetic segment classification	Accuracy, precision, recall, F1	Spatiotemporal modelling, feature fusion	Medium – phoneme discrimination support
Ribeiro et al. (2019)	CNN + DNN	TD (UltraSuite)	Raw UTI	Speaker-independent phoneme classification	Accuracy	Masked modelling for speaker generalisation	Medium – foundational speaker-independent modelling
Xu et al. (2024)	Masked Modelling + Hard Sample Mining	TD (UltraSuite)	Raw UTI	Phonetic segment classification	Accuracy	Self-supervised learning	Medium – enhances the scalability of phoneme classifiers
You et al. (2023)	Spatio-temporal masked autoencoder+ Token Shift Module	UltraSuite- UXTD	Raw UTI	Phonetic segment classification	Accuracy	Label-efficient SSL via mask modelling	Improves articulatory discrimination from raw UTI; a foundational step toward automated SSD screening
Dan et al. (2025)	Spatio-temporal masked autoencoder+ Token Shift Module	UltraSuite- UXTD	Raw UTI	Phonetic segment classification	Accuracy	Captures cross-frame dynamics via token shifting	Higher robustness for UTI classification; strengthens pipeline components needed for reliable SSD tools
Mozaffari and Lee (2019)	U-Net variant (BowNet)	Ottawa UTI Corpus	UTI Images	Tongue contour segmentation	Dice	Dilated CNNs for robust segmentation	Medium – real-time segmentation
Li et al. (2022)	wUNet (VGG-16)	NS, TJU, TIMIT	UTI	Tongue contour extraction	Intersection over Union (IoU)	Multi-dataset training, speaker-agnostic	Medium – segmentation across setups
Mukai et al. (2022)	U-Net-based contour point extraction	Institutional UTI	UTI frames	Tongue surface extraction	Accuracy	Quantifies impact of annotation design on learning	Supports efficient 3D tongue model construction
Zhao et al. (2019)	ConvLSTM	Silent Speech Dataset	UTI Video Sequence	Tongue motion prediction	Mean squared error (MSE)	Temporal dynamics captured	Low–indirect application to SSD
Cleland (2023)	N/A (Descriptive)	Clinical Case Series	UTI in Therapy	Qualitative evaluation of UTI use	Qualitative Insights	Real-world feasibility context	Highly informed design and deployment

## 3 Results

### 3.1 Corpora and datasets used in the included studies

A central limitation across the reviewed literature is the scarcity of large, well-annotated pediatric UTI datasets, especially for disordered speech. To make the landscape clear, Table 3 summarises the key corpora and datasets encountered in the included studies, and Table 4 maps each study to the dataset(s) it used. As shown, most classification studies rely on UltraSuite-UXTD (typically developing children), whereas resources featuring speech sound disorders (e.g., UltraSuite-UXSSD and UPX) are much smaller, narrower in error coverage, or require additional expert labelling per study. This imbalance motivates the recent adoption of self-supervised pretraining and class-imbalance handling (e.g., focal loss, hard-sample mining) to reduce annotation burden and improve robustness.

**Table 3:** Datasets used or referenced by the included studies.

Dataset	Population	N participants	Mean age	Language	Modality	Typical use in reviewed papers	Availability
UltraSuite-UXTD	TD children	58	~9y 3 m	Scottish English; clinic/school tasks	UTI + audio	4-way phoneme/gesture classification; pretraining	Public (UltraSuite)
UltraSuite-UXSSD	SSD children	8	~7y 7 m	Scottish English	UTI + audio	Error detection	Public (UltraSuite)
UltraSuite-UPX	Therapy (cleft palate ± cleft lip / SSD)	20	~8y 4 m	Therapy sessions	UTI + audio	Clinical/therapy	Public (UltraSuite)
Ottawa UTI Corpus	Mixed/TD (local)	n/a	n/a	Research lab	UTI	Tongue contour segmentation	Local / not public
NS, TJU, TIMIT-UTI	Mixed (lab)	n/a	n/a	Research lab	UTI	Tongue contour extraction	Local / not public
Silent Speech (WSJ0-derived; TJU)	Adults (lab)	n/a	n/a	Silent-speech interface	UTI video	Frame prediction / motion modelling	Local / not public
Institutional coronal UTI (Mukai et al., 2022)	Children (therapy-oriented)	19 cross-sections	n/a	Vowels	UTI	Tongue surface extraction for 3D modelling	Local / not public

**Table 4:** Mapping from included studies to dataset(s).

Study	Task	Dataset(s) used	Population
Ribeiro et al. (2021)	Error detection	UXTD (+ external adult TaL)	Children (TD + SSD)
Ani et al. (2024)	4-way phoneme classification	UXTD	TD children
Ribeiro et al. (2019)	Speaker-independent phoneme classification	UXTD	TD children
Xu et al. (2024)	Masked modelling + hard-sample mining	UXTD	TD children
You et al. (2023)	Self-supervised ViT; 4-way classification	UXTD	TD children
Dan et al. (2025)	Spatio-temporal masked modelling	UXTD	TD children
Mozaffari and Lee (2019)	Tongue contour segmentation	Ottawa UTI Corpus, Seeing Speech	Mixed
Li et al. (2022)	wUNet segmentation	NS, TJU, TIMIT-UTI	Mixed
Zhao et al. (2019)	Motion prediction	Silent-speech (WSJ0/TJU)	Adults
Cleland (2023)	Clinical/therapy context	Clinical case series	Children
Mukai et al. (2022)	Segmentation for 3D modelling	Institutional coronal UTI	Children

### 3.2 Overview of included studies

This review comprised a total of eleven studies in total. Despite their limited number, these studies represent the initial attempts to integrate DL and UTI to address speech production analysis and disorder diagnosis in children. Six investigations focused on direct SSD detection or associated phoneme categorisation, four on technical foundations, and one on the clinical use of UTI for disordered speech therapy. All included research utilised data from paediatric speakers, highlighting SSD’s paediatric focus; however, due to insufficient abnormal speech data, several studies substituted typically developing children as a replacement.

The research collectively represents elements of a potential end-to-end system, with some focusing on extracting usable features from raw ultrasound data and others attempting to classify those features into clinically significant outcomes. CNNs were the predominant architecture, often adapted for specific tasks. Two studies used recurrent or sequence models in conjunction with CNNs to handle the temporal dynamics of speech. The UltraSuite corpora and smaller lab-collected datasets are frequently utilised for specific tasks such as silent speech or tongue contour tracking. Due to the limited availability of accessible datasets, most studies have employed data augmentation or transfer learning to enhance performance, although model generalisability remains to pose a barrier, as elaborated below.

All studies presented various assessment metrics, often accuracy for classification tasks or Dice score/IoU for segmentation tasks, to illustrate feasibility. Direct comparisons between studies are problematic due to variations in tasks and datasets. The classification-focused studies achieved accuracy rates between 75 and 95% for their designed objectives, whilst the segmentation studies earned contour agreement scores ranging from the mid-80s to mid-90s, suggesting applicability in clinical settings. The singular clinical-focused investigation did not present quantitative metrics due to its observational nature. The subsequent subsections include a narrative synthesis of the findings from these investigations, arranged according to their placement in the proposed pipeline from ultrasound data to clinical outcomes.

### 3.3 DL applications in UTI for speech: from segmentation to disorder classification

#### 3.3.1 Automated classification of speech sounds and errors in children

A core motivation for applying DL to UTI is to automate the evaluation of whether a child produces a speech sound correctly or inappropriately, thereby supporting SSD diagnosis and therapy. In our review, six of the research analyses focused specifically on speech segment classification, proving that UTI can distinguish speech sounds and detect misarticulations.

For instance, Ribeiro et al. (2021) made significant efforts to identify SSDs utilising UTI. This study examined the application of UTI for the automated detection of speech articulation errors, concentrating on clinically relevant errors such as velar fronting and rhotic sound abnormalities in Scottish English-speaking children. To improve the system’s adaptability, it was trained using a combination of in-domain child speech data from the UltraSuite UXTD dataset and out-of-domain adult data from the TaL corpus. The evaluation of model performance utilised both typically developing and atypical speech samples. Experienced SLTs provided ground truth annotation evaluations based on synchronised ultrasound and audio recordings. There was significant inter-rater agreement in identifying velar fronting errors, but reduced consistency for rhotic errors.

The classification model, implemented as a CNN, utilised both ultrasound frames and corresponding audio features as input, with ultrasound data contributing significantly to the detection of place-of-articulation errors such as velar fronting. The algorithm achieved a maximum accuracy of 86.9% in classifying phonetic segments in typically developing child speech and accurately identified 86.6% of the velar fronting errors annotated by experts. Significantly, 73.2% of the errors detected by the system aligned with expert judgments, showing reasonably high precision. The findings on the detection of/r/−sound errors were less conclusive, most possibly due to poor inter-annotator agreement on those errors, suggesting the need for a more robust or objective annotation process for specific error types. Overall, this study demonstrates that UTI, when paired with DL, can be an effective technique for augmenting clinical speech evaluations. It demonstrated the viability of automatic detection of some speech errors, paving the way for the incorporation of automated error detection systems into speech treatment process, such as tracking intervention results in children with SSD.

Building on a similar technique but in typically developing speakers, Ani et al. (2024) focused on phoneme classification using UTI in developing children’s speech disorders. This study proposes a DL framework for the automatic classification of phonetic segments in child speech using raw ultrasound images. The method integrates visual and textual features obtained from the ultrasound. The aim was to enhance speaker-independent classification performance, which is generally challenging due to anatomical and speech variability.

Data were collected from the UltraSuite UXTD dataset, comprising UTI recordings from nine typically developing children. The study focused on utterances containing isolated words or non-words, categorising sounds into four principal phonetic classes based on place of articulation: (1) bilabial/labiodental, (2) dental/alveolar/postalveolar, (3) velar, and (4) the alveolar approximant (/r/). To generate texture information from the UTIs, the authors extracted features using the Local Binary Patterns (LBP) operator, which identifies local texture patterns and is extensively utilised in image analysis.

Several classification models were assessed, including a standard CNN, deep feed-forward neural networks (DNNs), and transfer learning using pre-trained image models (ResNet-50 and Inception-V3). Furthermore, Ani et al. (2024) proposed a novel dual-stream design named FusionNet. FusionNet has two parallel streams: one CNN-based stream processes the raw ultrasound image for shape-based features, while the other employs a fully-connected network to extract LBP texture features; these streams are subsequently integrated and jointly optimised to produce the final classification. The models were trained and evaluated under three conditions: speaker-dependent, where training and testing occurred on the same child, a multi-speaker scenario, involving training on many children and testing on a separate subset of those children; and speaker-independent, which entailed training on a group of children and testing on an entirely unseen child.

Experimental results indicated that FusionNet outperformed all other models. Specifically, FusionNet achieved a precision of 91.88% in the speaker-dependent scenario, 92.12% in the multi-speaker scenario, and 82.32% in the speaker-independent scenario. These findings demonstrate how combining complementary visual and texture characteristics can enhance the robustness and generalisability of UTI-based speech classification. The performance decrease in the speaker-independent scenario to approximately 82% indicates the challenge of generalising to new speakers. This study demonstrates that multi-modal learning has significant potential for improving UTI speech classification. This study focused on typically developing speech and phoneme classes; nevertheless, the developed methodology could be utilised in the future to classify specific speech error types, thereby enhancing SSD assessment tools.

Ribeiro et al. (2019) also contributed by investigating the challenges of speaker-independent phonetic segment classification using raw UTI from child speech. This study aimed to achieve the same objective as Ani et al. (2024) which was to improve generalisation across speakers. The authors developed a four-class classification task based on the place of articulation, utilising the UltraSuite UXTD dataset. The preprocessing methods were a notable aspect; they experimented with raw image normalisation and dimensionality reduction techniques, including principal component analysis (PCA) and the 2D discrete cosine transform (2D-DCT) on the ultrasound frames. These approaches aimed to reduce input size and eliminate certain speaker-specific characteristics, enabling the network to focus on essential features. They evaluated classification models, including feed-forward DNNs and CNNs, based on various input representations.

A key innovation was the utilisation of a speaker mean image, which accurately computes the average ultrasound frame for each speaker, captures that speaker’s typical tongue posture/background and provides that as an additional input channel to the CNN. The idea is that the network will learn to utilise this as a reference to normalise speaker-specific differences.

The results showed that in the absence of speaker adaptation, models performed significantly worse on unseen speakers. The CNN on raw images achieved approximately 67.0% accuracy in speaker-independent conditions, while in multi-speaker training, it reached ~74.8%. Interestingly, incorporating the speaker’s mean image as input improved performance, and using DCT-transformed inputs also gave competitive results, especially when training data were scarce. PCA-based input consistently underperformed the others in this context. Furthermore, Ribeiro et al. found that performing a small amount of speaker-specific modification significantly improved speaker-independent accuracy.

Overall, the findings highlight the challenges of generalising across unseen speakers in UTI-based speech classification, which is an important consideration for clinical use. They also demonstrate the effectiveness of speaker normalisation approaches and minimum adaptation in improving robustness. This study provides important foundational insights for developing models capable of handling the broad anatomical and speech variability observed in children, particularly those with SSD.

A notable advancement of the masked modelling paradigm was presented by Xu et al. (2024) and You et al. (2023). This study proposed a self-supervised learning framework to classify phonetic segments from raw UTI data to improve performance in low-data scenarios. Their approach employed masked image modelling and hard sample mining rather than requiring significant labelled data. The objective was to train a model to reconstruct missing parts of the ultrasound image so that it learns robust features of tongue shapes without needing labels. After this pre-training, the model is fine-tuned for phoneme classification. They employed a hard sample mining strategy where difficult frames, those near phoneme boundaries, which are often misclassified, were enhanced to enhance the model’s ability to manage challenging frames during training.

Evaluated on the UltraSuite typical developing dataset, their model achieved phoneme classification accuracies of over 85%, representing a significant improvement over many prior results, especially in scenarios with limited annotated data. This study underscores the scalability of DL models with minimal supervision, a key consideration for clinical translation where large, labelled datasets of disordered speech are scarce. By leveraging unlabelled data, the approach by Xu et al. (2024) strengthens the case for self-supervised learning as a path toward robust, annotation-efficient articulatory models. This method could help future SSD detection systems train on a wealth of unlabelled ultrasound data to improve their feature extraction, therefore requiring only a smaller size of labelled disordered data to achieve optimal performance.

A notable extension of the masked-modelling paradigm was introduced by You et al. (2023), who framed phonetic segment recognition from raw midsagittal UTI as a self-supervised pretrain to the supervised fine-tune problem. Their approach pretrains a vision transformer (ViT) encoder by masked image modelling on a large amount of unlabelled UTI, encouraging the network to reconstruct withheld patches and thereby internalise robust articulatory structure without labels. The pretrained encoder is then fine-tuned for 4 phoneme classification on UltraSuite-UXTD, evaluated across dependent, multi-speaker, independent, and adapted scenarios. Reported accuracies were 88.10, 84.82, 83.72, and 88.94%, respectively, amounting to an average +13.3% gain over a SimSiam baseline. In practical terms, this study highlights how self-supervised pretraining on unlabelled UTI can significantly decrease dependence on scarce annotations while enhancing robustness across speakers and sessions, an important step toward annotation-efficient pipelines in clinical SSD applications.

Building on this line of work, Dan et al. (2025) advance masked modelling into the spatio-temporal domain, arguing that reliable phonetic discrimination in UTI requires modelling frame-to-frame articulatory dynamics. They employ a ViT-based spatio-temporal masked autoencoder augmented with a token shift module to propagate information across adjacent frames during pretraining, followed by supervised fine-tuning for 4-way classification on UltraSuite-UXTD. The model achieves an accuracy of 90.32% for dependent, 86.45% for multi-speaker, 85.27% for independent, and 90.11% for adapted accuracy, with performance remaining stable even at high masking ratios of ≈75%. By explicitly capturing temporal structure under limited labels, this study shows how motion-aware self-supervision can further enhance generalisation and label efficiency, narrowing the gap between research-grade UTI classifiers and clinically robust articulatory recognition needed for SSD screening and therapy support.

In summary, these six studies trace a clear trajectory: from clinical error detection (Ribeiro et al., 2021) through TD phoneme/gesture classification (Ani et al., 2024; Ribeiro et al., 2019) to annotation-efficient self-supervised modelling (Xu, 2024; You et al., 2023; Dan et al., 2025). CNN-based pipelines and their transformer-based extensions consistently separate UTI-encoded articulations, but generalisation to unseen speakers and error types with low annotation reliability remains an open challenge. Promising strategies include speaker normalisation/adaptation, multi-modal inputs (ultrasound + audio), and self-supervised pretraining that exploits large stores of unlabelled UTI, practical steps toward deployable, clinician-supportive tools for SSD assessment and monitoring.

#### 3.3.2 Foundational tools: tongue segmentation and motion modelling

Beyond classification, several studies have concentrated on foundational technical tasks that are essential for a fully automated analysis pipeline. Chief among these is tongue segmentation, the automatic identification of the tongue surface in each ultrasound frame and motion modelling, which captures dynamic tongue movement and has implications for silent speech interfaces and articulatory analysis.

Two of the reviewed studies addressed the longstanding challenge of automatic tongue contour extraction using DL. Accurate tongue segmentation is critical because it transforms raw ultrasound images into sequences of structured tongue shapes, which can then be further analysed or fed into classification models. Traditional methods like EdgeTrak often struggled with noise and often required manual correction, but DL offers a data-driven solution with improved generalisability and automation.

Mozaffari and Lee (2019) introduce BowNet and wBowNet, two novel deep CNN architectures designed for fully automatic and real-time tongue contour extraction from UTI. Recognising the challenges posed by the noisy, low-contrast nature of UTI, the authors designed these models to capture both local and global contextual information through a combination of standard and dilated convolutions. The networks operate end-to-end, with an encoder-decoder structure inspired by UNet, and DeepLab v3, using a VGG-16 backbone. The *wBowNet* variant features a more deeply interwoven architecture to enhance feature resolution and context at multiple scales.

The models were trained and validated on two datasets, a local University of Ottawa UTI dataset and the publicly available Seeing Speech dataset. They employed both online and offline data augmentation to increase robustness. Notably, the authors also developed a Python-based annotation tool utilising B-spline interpolation to produce smooth ground truth contours from manual points, addressing inconsistencies in manual labels and ensuring high-quality training data.

Extensive evaluations showed that both BowNet variants achieved robust and accurate segmentation. On cross-validation within and across datasets, *wBowNet* slightly outperformed BowNet, with mean Dice scores around 0.85 when evaluating tongue boundary overlap with ground truth. Under cross-dataset validation, performance understandably dropped but remained quite excellent, indicating some generalisation. Importantly, both models maintained real-time performance on a GPU, and their compact architecture meant they used less memory than a standard U-Net with similar accuracy. Overall, BowNet and wBowNet represent a significant advance in UTI segmentation, offering a scalable and relatively accessible tool for researchers and potentially for clinicians to automatically track tongue movements. This is an enabling technology by reliably extracts tongue contours from ultrasound, and subsequent classification of speech sounds or visual feedback in therapy becomes more feasible.

Building on similar concepts, Li et al. (2022) proposed wUnet, an enhanced CNN architecture tailored for tongue contour extraction, particularly in the context of silent speech recognition. wUNet extends the U-Net framework by adding extra skip connections between encoding and decoding layers and by using a VGG-16 network to initialise the encoder. Additionally, it includes a multi-level feature fusion strategy to combine feature maps from different depths, presumably to better capture both low-level edge information and high-level shape information.

Li et al. trained and evaluated wUNet on three datasets: the NS dataset, the TJU dataset, and the TIMIT UTI dataset. They compared wUNet against baseline models like a vanilla U-Net and UNet++. The results were impressive, wUNet outperformed both U-Net and UNet++ in segmentation accuracy. For example, on the NS dataset, wUNet achieved a peak IoU of 98.22% and a Dice coefficient of 94.47%, substantially higher than baseline models. It also showed lower sensitivity to image resolution differences and training data volume, indicating strong generalisability and efficiency.

These results affirm wUNet’s potential for real-time, high-precision tongue tracking. High IoU (~98%) implies that the predicted tongue contours almost perfectly overlapped the manual contours, which is near-human performance. Its robustness across multiple datasets suggests it could handle different ultrasound machines or populations. While this study framed the work in the context of silent speech interfaces, the ability to accurately and automatically extract tongue contours has direct relevance for clinical tools as well, since those contours can be used for visual biofeedback or as input to classification algorithms for error detection.

Mukai et al. (2022) add a complementary perspective by examining how annotation design affects learning for tongue surface extraction aimed at 3D tongue modelling. Using an institutional dataset (19 coronal cross-sections; 264 base images expanded to ~7,700 via augmentation; 44 test images), they compared teachers defined by sparse points versus splines and trained a U-Net-based contour-point extractor. With spline-based teachers, the model achieved 91.7% horizontal multiplicity, 4.1 px relative vertical error, and 81.8% subjective acceptability, with vowel-wise error profiling. This careful quantification shows that labelling protocol choices materially influence segmentation quality, offering practical guidance for building efficient training sets and for downstream editable 3D models of the tongue, directly relevant to clinical scenarios such as lateral misarticulation therapy planning.

A complementary technical advancement was proposed by Zhao et al. (2019) and Zhao et al. (2019), who investigated convolutional long short-term memory (ConvLSTM) networks for predicting tongue motion in unlabelled UTI sequences. Unlike the previous segmentation works that focus on static frame-by-frame analysis, this study addresses dynamic modelling. The task was to predict future ultrasound frames given a sequence of past frames. This was done in the context of a silent speech interface, but the approach is generally applicable to articulatory motion prediction. They trained a ConvLSTM model to predict the next few frames of a UTI video based on the preceding 8 frames. Two datasets were used, one derived from the WSJ0 speech corpus and another from TJU. Performance was assessed using metrics like MSE between predicted and actual frames, and a structural similarity metric (CW-SSIM) adapted to evaluate how well the motion was captured.

The ConvLSTM consistently outperformed a 3D-CNN baseline. It was also able to maintain reasonable accuracy for several frames. Notably, while ConvLSTM excelled at raw pixel prediction, when it came to directly predicting contours, a 3D CNN was slightly better for that specific task, suggesting the ConvLSTM might smooth out some high-frequency detail. Nonetheless, the ConvLSTM captured the temporal dynamics of tongue movement with high fidelity.

For clinical relevance, a model like this could be used to anticipate articulatory movements or to detect anomalies in motion. It could also be part of a system providing real-time feedback, for instance, predicting where the tongue should go next, to compare against where it does go in a child with apraxia. While Zhao et al.’s application was silent speech, their approach underscores the value of sequence models in capturing coarticulation and speech dynamics, which are very relevant for assessing certain speech motor disorders.

In summary, these segmentation (Mozaffari and Lee, 2019; Li et al., 2022; Mukai et al., 2022) and motion-prediction (Zhao et al., 2019) studies provide the building blocks for ultrasound-based speech analysis. Automatic, high-quality contouring reduces manual effort and converts UTI into structured articulatory representations; sequence models capture how these shapes evolve. Together they ease persistent barriers, speckle noise, labelling burden, and temporal complexity, and move the field toward a fully automated pipeline in which high-level articulatory information is extracted reliably and made available for downstream error detection, therapy monitoring, and clinician-facing biofeedback.

#### 3.3.3 Clinical insights: ultrasound in practice for SSD therapy

While most of the included studies focus on algorithmic developments, one study by Cleland (2023) provides crucial clinical insights by examining the use of UTI in both research and therapeutic practice for individuals with cleft lip and palate (± cleft lip) is a condition often associated with compensatory articulatory strategies and persistent SSD, making it an important test case for ultrasound feedback.

Drawing on case examples, Cleland (2023) describes how UTI is used as a visual biofeedback tool to support articulation therapy post-palate repair. One key insight from this case series is that UTI can reveal atypical tongue movements and covert contrast errors that are not apparent through audio-based assessment alone. For example, children with cleft-related SSDs may exhibit abnormal articulatory placements such as posterior or double articulations, which are often difficult to discern by ear but can be visualised and directly addressed in therapy with ultrasound. Real-time ultrasound images allow therapists to see where the tongue is making contact or forming constrictions, thus helping them guide the child to a more typical articulation.

Cleland reported that using UTI in therapy improved some children’s awareness of their tongue placement and helped in correcting misarticulations that had been resistant to change. However, the study also notes several limitations of the current clinical use of UTI. Interpretation of ultrasound images requires substantial expertise. Typically, a clinician must analyse the images in real-time and provide verbal guidance because the child cannot interpret the ultrasound screen by themselves. This is precisely where automation could provide significant support, for instance, a system that could automatically detect and highlight certain articulatory features in real time would offload some cognitive work from the clinician and provide more direct feedback to the patient.

Another practical consideration mentioned is the need for specialised equipment and training. While UTI is non-invasive and child-friendly, not all clinics have ultrasound machines or clinicians trained to use them for speech therapy. Therefore, evidence from Cleland’s work helps identify what would make UTI more viable clinically. For example, simplified user interfaces, automated annotation, and perhaps some quantification of progress. Such features correspond to the technical developments that the other studies are working toward.

In conclusion, the reviewed studies together outline a path toward automated SSD detection and feedback using UTI. The initial step is ensuring the reliable extraction of articulatory data, like tongue contours from ultrasound images. This data can then be used to classify speech sounds and detect articulatory errors. Ultimately, these tools must be embedded into clinical workflows to be truly useful. The results so far demonstrate high accuracy in controlled experiments and show strong clinical relevance, but they also highlight that the field is in its early stages, most models have been evaluated on limited datasets or in lab settings.

The next section explores how these foundational elements can be integrated into a coherent pipeline, the challenges that remain, and the research directions needed to bring this technology into everyday clinical practice.

## 4 Discussion

### 4.1 Toward an integrated DL pipeline for SSD detection

**Figure 4:**
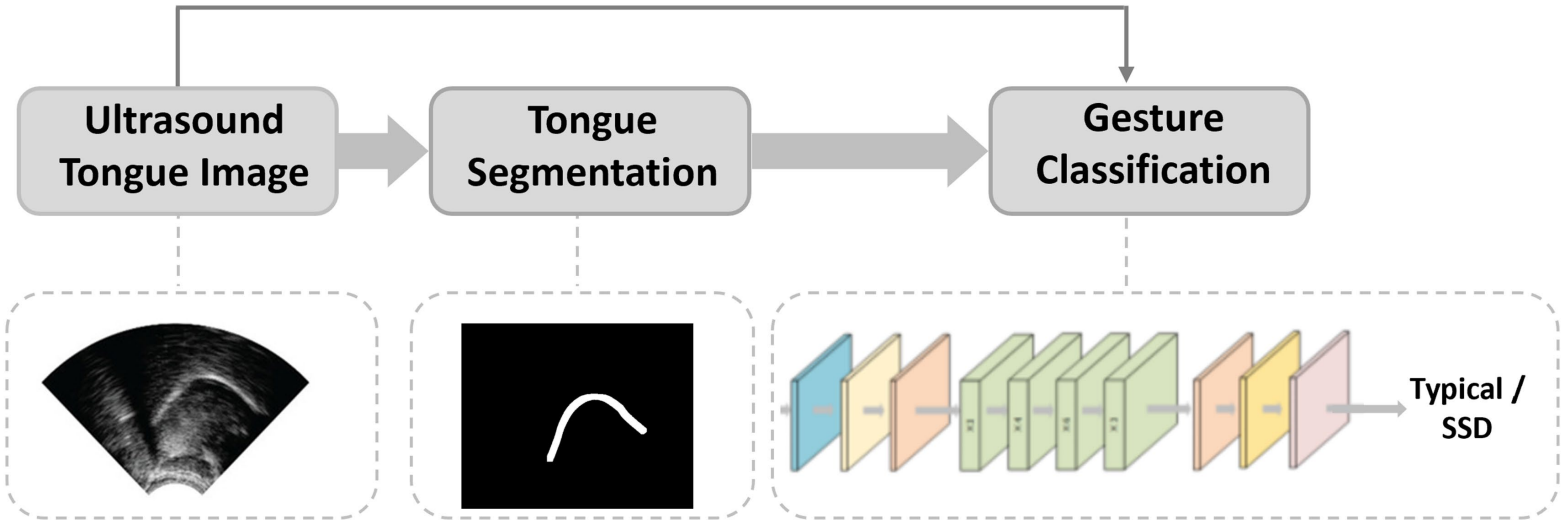
DL pipeline for SSD detection.

The literature collectively outlines a practical pipeline for automated detection of SSD with UTI and DL. No single study implements the entire pathway end-to-end, but the reviewed works provide complementary advances that illuminate the route to clinical integration. Figure 2 gives a high-level overview of the end-to-end system. Figure 4 illustrates representative outputs at each stage of the technical pipeline.

The pipeline typically begins with preprocessing and segmentation to isolate the tongue surface and reduce speckle/artefacts. DL contour extractors such as BowNet/wBowNet (Mozaffari and Lee, 2019) and wUNet (Li et al., 2022) demonstrate accurate and robust tongue boundary extraction. Recent work by Mukai et al. (2022) shows that annotation design (points vs. spline teachers) materially impacts segmentation quality for 3D tongue modelling, offering practical guidance for dataset curation. At the same time, several classification studies operate directly on raw UTI without explicit segmentation, indicating two viable architectural paths: segmentation-first when interpretable contours are needed, and raw end-to-end when throughput and label-efficiency dominate. In the second stage, the sequences of tongue shapes are analysed for phoneme classification or speech error detection. Foundational TD classification studies (Ribeiro et al., 2019; Ani et al., 2024) highlight both the promise of UTI-based articulatory cues and the challenge of generalising to unseen speakers, mitigated by speaker normalisation/adaptation and multi-modal inputs of ultrasound and audio. Clinically targeted work (Ribeiro et al., 2021) shows that UTI materially aids the detection of place-of-articulation errors of velar fronting, though reliability for/r/remains limited by annotator agreement, underscoring the need for objective targets/labels for certain error types.

A parallel, rapidly developing studies uses self-supervised masked modelling and Transformer encoders to improve label efficiency and cross-speaker robustness on raw UTI. Xu et al. (2024) demonstrated masked-image pretraining with hard-sample mining. You et al. (2023) pre-trained a ViT on unlabelled UTI and fine-tuned for 4-way phoneme classification, reporting +13.3% over a SimSiam baseline. Dan et al. (2025) extended masked modelling to the spatio-temporal domain with a Token-Shift module, achieving high accuracy while remaining stable at high masking ratios of ~75%. Collectively, these results indicate that SSL and Transformers are strong candidates for annotation-efficient pipelines that must generalise across children, sessions, and devices.

A parallel stream in the pipeline is motion modelling, which captures dynamic tongue behaviour relevant to motor-speech disorders. ConvLSTM-based predictors (Zhao et al., 2019) forecast future frames with high fidelity, suggesting uses in anticipatory feedback and trajectory consistency assessment, even if 3D-CNNs may retain an edge for sharp contour prediction. Integrating motion cues with static classifiers is a natural next step for nuanced SSD assessment.

The final stage involves translation of model outputs into clinically meaningful feedback or measures. This could include converting detected phonemes or errors into summary reports for SLTs or providing real-time visual or auditory biofeedback to clients during therapy. This stage also encompasses user interface design, integration into clinical workflows, and ensuring the system can operate in real-time during a therapy session.

Importantly, the pipeline framework underscores the tension between technical capability and clinical applicability. For example, a high-accuracy segmentation algorithm could be of limited use if it cannot run in real time or if its output is not interpretable by clinicians. Similarly, a phoneme classifier trained exclusively on typical speech may perform poorly when applied to disordered populations, where articulatory patterns diverge significantly. This reinforces the notion that technical development must be guided by clinical objectives, not merely optimised in a vacuum. To be useful, each stage of the pipeline must consider end-user requirements: speed, accuracy across diverse populations, and transparency.

Encouragingly, some studies already bridge multiple stages. Ribeiro et al. (2021), for instance, implicitly combined segmentation and classification to detect articulatory errors from raw UTI frames. Their work hints at an end-to-end approach where the system goes straight from ultrasound to an error decision. This type of integration is promising and demonstrates the feasibility of building more comprehensive systems.

Overall, the reviewed literature demonstrates solid progress in the early stages of the pipeline and emerging work on dynamic modelling. There has been limited exploration of the final integration stage, with Cleland (Cleland, 2023) being an initial foray into that. Current systems remain largely task-specific, trained on constrained datasets, and rarely validated in real-world clinical environments. Future research must focus on bridging these gaps, developing integrated, interpretable, and real-time systems that meet clinicians’ practical needs and directly enhance therapeutic outcomes for individuals with SSD.

### 4.2 Challenges faced by DL techniques in analysing speech problems

Despite the promising developments outlined in this review, several challenges and gaps must be addressed to implement these DL techniques in standard clinical practice for SSD detection. We identify the principal challenges as follows:

- Limited and unbalanced data: A primary challenge is the scarcity of annotated UTI data for disordered speech. Only a few small datasets exist specifically for SSD, for example, the UltraSuite-SSD includes data from only a handful of children and covers only a limited range of speech errors. Most DL models have therefore been trained on typically developing speech or on very limited disordered samples. The scarcity of data and class imbalance hinder networks from acquiring disorder-specific articulatory patterns, frequently resulting in overfitting and poor generalisation (Al-hammuri et al., 2025). One review notes that models frequently excel on existing test data but fail on unseen speakers or settings, largely due to limited training datasets and insufficient diversity. Enhancing datasets via augmenting or cross-domain transfer is regarded as crucial for improving robustness (Al-hammuri et al., 2022).
- Speaker variability and generalisability: UTI data significant inter-speaker variability. Anatomical variances, such as tongue length, palate shape, probe placement variations, and differences in speaking style, all influence the ultrasound images. Numerous models in the reviewed studies exhibited strong performance in within-corpus evaluations; nevertheless, a model trained on one group often encounters difficulties when applied to a different group. Ribeiro et al. (2019) observed a significant decline in accuracy for unseen speakers, despite using normalisation techniques. The generalisation issue is critical; an SSD detection system may perform effectively on the research team’s dataset but may fail when implemented in a new clinic with different equipment or patients. Addressing this issue may require robust data augmentation, domain adaptation methodologies, or training on considerably more diverse data. The variability is even greater in disordered speech, as each child’s compensatory articulation can be unique. Therefore, ensuring that models are speaker-independent or can quickly adapt to a new speaker poses a significant challenge for practical application.
- Quality of ultrasound image and noise: The ultrasound modality presents technical challenges for DL. UTI frames are often low-contrast, noisy images with speckle artefacts and occasional shadowing or occlusions (Song et al., 2024; Xia et al., 2024). This image quality hinders feature extraction and model learning. Standard computer vision techniques struggle with the lack of clear edges or consistent textures in UTI. Even advanced CNN-based segmentation models must contend with speckle noise and varying brightness, which can degrade accuracy. Enhancing the image quality through better preprocessing, denoising, or novel ultrasound hardware and designing noise-robust architectures is a significant focus of current research to tackle this issue.
- Interpretability of models: Current UTI-based models, like many other DL systems in medicine, frequently function as “black boxes,” providing limited details about the decision-making criteria. Clinicians may be reluctant to rely on automated judgments on speech errors without clear rationales. However, deep CNN or Transformer models for ultrasound are complex and not easily interpretable, especially when trained on limited, noisy datasets. The absence of transparency is compounded by the variability of input data, and subtle tongue shape characteristics acquired by the network are not intuitively understood by human experts. Recent studies highlight that the intricacy of these models, combined with data limitations, makes them difficult to understand and prone to unexpected or irrelevant outputs. This highlights the need to integrate explainable AI techniques or more interpretable model architectures to ensure that speech-language pathologists can trust and effectively use the outputs in diagnosis. For example, visualising which part of the ultrasound image influenced a decision, such as saliency mapping or providing a clear measure, like tongue curvature, might bridge this gap.
- Restricted clinical validation and integration: The most significant gap is the limited involvement of clinicians and patients in developing and testing these systems. The literature consists mainly of engineering-driven studies assessing models on research datasets, with almost no trials of a speech therapist using a DL-powered UTI system in real clinical settings. Consequently, usability issues remain unaddressed; in the absence of end-users’ feedback, current prototypes may miss practical requirements. Moreover, models validated only on ideal datasets may not perform well in noisy clinical environments. Integrating UTI analysis into real-time therapy sessions and demonstrating effectiveness in improving clinical results presents significant obstacles.
- Absence of standardisation in evaluation: Presently, there is a consensus on evaluation protocols. Diverse studies employ distinct datasets, target tasks, and performance metrics, which makes it difficult to compare results or track progress (Al-hammuri et al., 2022). Segmentation studies may present Dice scores or mean distance errors, while classification studies report accuracy or F1 scores, often on different speech targets. Unlike other fields of speech technology, there is no standardised benchmark dataset for UTI-based speech assessment. The lack of standardised evaluation criteria implies that reported performance may not be comparable between studies or reflect clinically relevant outcomes. Establishing unified datasets that encompass a diverse range of disordered and typical cases, along with standardised metrics, will facilitate the community in systematically assessing advancements and ensuring that algorithms fulfil clinical requirements.

### 4.3 Recommendations for future research

To advance toward clinically implemented methods for SSD detection utilising DL and UTI, we propose several priorities for future research, each directly addressing the challenges above:

1. Expand datasets of disordered UTI speech. The community needs to develop and release larger, more diverse UTI datasets that specifically include children with SSD. This initiative could resemble the advantages gained by the speech recognition domain through the utilisation of shared corpora. The new data should include a variety of SSDs and detailed annotations. The new data must encompass a diverse range of ages and severities, enabling models trained on it to acquire robust features. Collaboration with hospitals and clinics can help gather such data ethically and efficiently. Recent medical-imaging surveys highlight the rapid adoption of transformers and self-supervised learning, suggesting label-efficient pretraining and hybrid CNN-Transformer decoders could mitigate small, labelled UTI datasets (Huang et al., 2022; Shamshad et al., 2023).
  By sharing these datasets openly, researchers can benchmark their models on common test sets, accelerating progress.
2. Enhance model generalisability: Future models should be designed with cross-speaker and cross-domain generalisation. Techniques like domain adaptation (Guan and Liu, 2022), data augmentation (Chlap et al., 2021; Kumar et al., 2025), and multi-task learning are promising. Additionally, implementing a method of speaker normalisation, such as calibrating a model with limited samples from a new speaker, could boost practical performance.
3. Integrate and optimise pipelines for real-time application: Future efforts should focus on combining DL models into end-to-end systems that operate in real time. A pipeline could use a segmentation CNN to preprocess each frame and then feed it into a classification model to detect errors. Such integration must be optimised for speed to give instant feedback during therapy. Researchers should evaluate these pipelines holistically and ensure the overall system’s output remains accurate and intelligible to clinicians. Ultimately, the presentation of a fully integrated prototype in a clinical or realistic environment would be a major milestone.
4. Establish standardised evaluation benchmarks: The discipline would benefit from uniform evaluation criteria. This entails the development of standardised test datasets and reporting clinically relevant metrics. In addition to accuracy or Dice scores, studies should report metrics like per-phoneme recall for error detection. Furthermore, subsequent research should uniformly disclose inference time, model size, and the criteria for error definition to facilitate comparison. Utilising a standardised assessment terminology enables researchers to more efficiently refine systems to achieve the requisite performance for clinical use.
5. Enhance clinical collaboration and validation: Interdisciplinary collaboration with SLTs and clinical researchers should be intensified. User-centred design principles can ensure that the outputs of these DL models align with what clinicians find useful. Conducting trials in clinical environments, including minor feasibility studies, is essential for subsequent progress. These studies could involve an SLT using a prototype system during sessions and providing qualitative feedback on its utility or measuring outcomes like reduced assessment time or improved accuracy of diagnoses with the tool. Additionally, involving clinicians in the training loop could open paths for online learning or refinement of models in deployment. Ultimately, demonstrable evidence of enhanced patient outcomes or increased efficiency from utilising a DL-augmented ultrasound system will be required to substantiate clinical adoption. Involving stakeholders early will facilitate the eventual integration of this technology into everyday practice.

By implementing these recommendations, developing different datasets, building more generalisable and integrated models, standardising evaluations, and validating in real-world settings, the field can accelerate toward its objectives. Each recommendation addresses a current weakness; increased data and collaboration will mitigate data scarcity; robustness techniques will address variability; integrated pipelines will advance us from isolated demonstrations to comprehensive solutions; standards will guarantee consistent progress measurement; and clinical validation will maintain the relevance of the work within actual patient care. This aligns with the broader trend in health technology toward translational engineering, turning promising prototypes into effective tools that improve healthcare delivery.

## 5 Conclusion

This systematic review examined the emerging intersection of DL and UTI, emphasising their combined potential to automate the detection of SSDs. We identified and synthesised eight key studies that collectively illustrate the current advancements, some developed direct classifiers for speech errors or phoneme production, others provided enabling technology such as tongue segmentation and motion modelling, and one highlighted how ultrasound is currently used in therapy. Together, these works demonstrate that DL algorithms can extract clinically meaningful information from raw ultrasound of the tongue, from classifying fine-grained phonetic details in typically developing speech to detecting misarticulations in children with SSD. Importantly, real-time capable models such as CNNs and LSTMs have achieved accuracy levels that approach practical usability, at least within controlled settings.

Despite the relatively small number of studies, the narrative of technological evolution is clear. Early efforts focused on demonstrating feasibility. Subsequent works have improved robustness and scope, such as moving from single-speaker models to speaker-independent ones, and from static image analysis to spatial–temporal modelling. Moreover, the integration of these techniques is on the horizon: the concept of an end-to-end pipeline that takes raw ultrasound and outputs a diagnostic aid is now much more tangible than it was a few years ago. Each evaluated study has contributed a piece of this puzzle, whether through a novel network architecture or empirical evidence regarding the information UTI can offer.

Simultaneously, our evaluation highlights that key challenges remain unresolved. The primary requirement is the acquisition of comprehensive, high-quality data of disordered speech to effectively train and test these systems in ecologically valid ways. Many studies depended on data from typically developing speakers or very limited disorder datasets, which raises questions about how well the findings generalise. A further problem is achieving consistency and generalisability: a model that performs in one research lab may fail in a different clinic environment due to differences in equipment or patient demographics. There is also a translational gap between algorithm performance to actual clinical impact. No study has yet completed the cycle by deploying a DL-UTI system in live therapy sessions and assessing outcomes, an essential step for proving the technology’s efficacy in practice.

In conclusion, although the use of DL for UTI diagnosis for SSD is developing, the existing evidence is promising. We now possess a proof-of-concept demonstrating that non-invasive, real-time imaging of the tongue, combined with advanced AI algorithms, can detect speech sound errors that may elude human auditory perception or provide objective validation of hypothesised articulatory patterns. Ultimately, these advancements could lead to a new generation of clinical tools. This integration of engineering and healthcare exemplifies translational innovation by transforming technology advancements into practical benefits for those with communication disorders. Through sustained interdisciplinary collaboration, the prospect of an AI-assisted ultrasound system integrating into routine speech therapy is imminent, offering enhanced timeliness, precision, and efficacy in the care for children with SSD.

## Data Availability

Publicly available datasets were analyzed in this study. This data can be found at: https://ultrasuite.github.io/.
